# Targeting the PI3K/Akt/mTOR Pathway – Beyond Rapalogs

**DOI:** 10.18632/oncotarget.188

**Published:** 2010-10-22

**Authors:** Ben Markman, Rodrigo Dienstmann, Josep Tabernero

**Affiliations:** ^1^ Centre for Cancer Research, Monash Institute of Medical Research, Southern Health, Melbourne, Victoria, Australia; ^2^ Medical Oncology Department, Vall d'Hebron University Hospital, Barcelona, Spain

**Keywords:** Rapalog, PI3K, Akt, mTOR, cancer, inhibitor, clinical trial

## Abstract

It is well established that the PI3K pathway plays a central role in various cellular processes that can contribute to the malignant phenotype. Accordingly, pharmacological inhibition of key nodes in this signaling cascade has been a focus in developmental therapeutics. To date, agents targeting upstream receptor tyrosine kinases are best studied and have achieved greatest clinical success. Further downstream, despite efficacy in certain tumor types, the rapalogs have been somewhat disappointing in the clinic. Novel inhibitors of PI3K, Akt, and mTORC1 and 2 are now passing through early phase clinical trials. It is hoped that these agents will circumvent some of the shortcomings of the rapalogs and lead to meaningful benefits for cancer patients.

## INTRODUCTION

The PI3K (phosphatidylinositol 3-kinase) pathway is a signal transduction cascade that is central to a variety of important physiological functions, including cell cycle, cell survival, protein synthesis and growth, metabolism, motility and angiogenesis. Constitutive pathway activation, which occurs in human cancer at considerable frequency due to a variety of genetic aberrations, can induce a malignant phenotype by contributing to the hallmarks of cancer. Many small molecule inhibitors targeting key nodes in the pathway – PI3K, Akt and mTOR (mammalian target of rapamycin) – are at various stages of clinical development. Clinical experience is adding to the preclinical knowledge base regarding these agents, broadening not only the understanding of the similarities and differences between the compounds, but also of the machinations of the pathway itself. In this review we will focus on the development of those inhibitors that have reached clinical evaluation and how their future use may evolve.

## THE PI3K/AKT/MTOR PATHWAY

The PI3Ks are a family of lipid kinases that share the primary biochemical function to phosphorylate the 3-hydroxyl group of phosphoinositides [1]. Three classes (I-III) of PI3K are described that vary in structure and substrate preference. The heterodimers that make up class I PI3Ks consist of a regulatory and a catalytic subunit. In the class IA group, these are p85 and p110 (α, β and δ), respectively, whereas the class IB PI3K consists of p101 and p110γ [2]. Class II PI3Ks are monomeric catalytic isoforms, and the sole class III member is Vps34.

Isoform-specific functions of the class I PI3Ks are described, albeit with some redundancy, with potential implications for toxicity and efficacy of novel inhibitors of this class [3]. In broad terms, the ubiquitously expressed p110α and p110β influence cellular proliferation and insulin signaling, whereas p110γ and p110δ, primarily expressed in leukocytes, appear involved in immune function and inflammation. Class II PI3Ks assist in the regulation of membrane trafficking and the class III PI3K is involved in autophagy [4]. Class IA PI3Ks are implicated in human cancer.

Upstream receptor tyrosine kinases (RTKs) that feed into the PI3K pathway include members of the human epidermal growth factor receptor family (EGFR and HER2), platelet derived growth factor receptor, and the insulin and insulin-like growth factor 1 (IGF-1) receptors. Engagement of a growth factor with its RTK is the typical initiating event for activation of class IA PI3Ks, where RTK stimulation leads to an interaction with p85 in the tyrosine kinase domain. This can occur either directly (such as with HER3) or indirectly via adaptor molecules (such as the insulin receptor substrate 1, IRS1). Binding removes the inhibitory effect of p85 on p110, resulting in full activation of PI3K. The activated kinase converts its substrate phosphatidylinositol 4,5-biphosphate – PI(4,5)P2 – into PI(3,4,5)P3. PI(3,4,5)P3 (or PIP3) acts as a docking site bringing Akt and PDK1 into close proximity, allowing the latter to phosphorylate Akt at threonine-308 in its kinase domain. The mTOR-rictor complex (mTORC2) also contributes a phosphate group to Akt, at serine-473 in its helical domain. Both events are necessary for full Akt activity [5].

**Figure 1:**
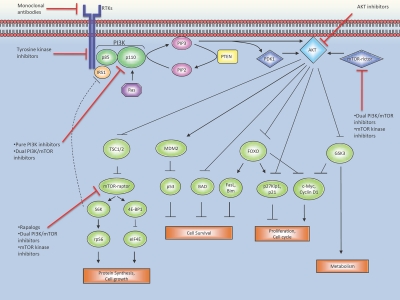
The PI3K/Akt/mTOR signaling pathway and associated inhibitors A ligand engaged RTK binds PI3K, either directly or indirectly via adaptor molecules such as IRS1, removing the inhibitory action of the p85 regulatory subunit on the catalytic p110 subunit. The active kinase generates PIP3 at the lipid membrane. PIP3 facilitates the phosphorylation of Akt by PDK1, while the mTOR-rictor complex contributes a second phosphate residue to Akt. As the central effector of the PI3K pathway, Akt transmits signal to a host of downstream substrates, thus influencing a variety of key cellular functions. Pathway activity is negatively regulated by PTEN and the S6K-IRS1 feedback loop. Pharmacological inhibition of the pathway is achieved through a variety of compounds in clinical use at various points along the pathway that are indicated by the red ⊣.

Akt, a serine/threonine kinase, is the central mediator of the PI3K pathway with multiple downstream effectors that influence key cellular processes (see figure 1). Akt stimulates protein synthesis and cell growth by activating mTOR (as part of the mTOR-raptor or mTORC1 complex) through effects on the intermediary tuberous sclerosis (TSC) 1/2 complex. It influences cellular proliferation by inactivating cell cycle inhibitors (p27 and p21) and promoting cell cycle proteins (c-Myc and cyclin D1) [6,7]. Akt mediated inhibition of pro-apoptotic genes (BAD and BIM) and degradation of the tumor suppressor protein p53 limits programmed cell death and enhances cell survival [4]. PI3K also features in cellular metabolism and insulin signaling through actions on GSK3 [8].

PI3K pathway activity can be switched off through the action of various proteins. The SHIP phosphatases abrogate signaling by converting PIP3 into the alternate PI(3,4)P2. A second mechanism involves the PTEN (phosphate and tensin homologue deleted on chromosome ten) tumor suppressor, a dual specificity phosphatase that dephosphorylates both protein and lipid substrates. Importantly, PTEN antagonizes PI3K function and negatively regulates Akt activities by stripping a phosphate off PIP3 thereby returning it to its original PI(4,5)P2 form.

Finally, S6K (one of the key effectors of mTOR) can feedback to downregulate IRS1, the adaptor molecule linking the IGF-1 receptor and PI3K. This effect appears to be direct and to impede the ability of IRS1 to associate with the insulin receptor. The outcome is to dampen further input into the PI3K pathway in the presence of ongoing stimulation of the insulin/IGF-1 receptors [9].

In addition to the complexity of the PI3K pathway, extensive crosstalk exists with other cellular signaling networks. For example, mTOR exerts influence on PI3K signaling via the S6K-IRS1 feedback loop and via mTORC2 mediated Akt-Ser473 phosphorylation [5,10]. Activation of the tumor suppressor p53 causes both increased PTEN and decreased p110 expression. Further, p53 degradation is reduced indirectly by PTEN via its antagonism of PI3K [11,12]. These actions safeguard the cell in times of genotoxic strain against ongoing DNA replication, though the interplay between p53 and PTEN requires further elucidation. Finally, activated GTP-bound RAS proteins are capable of activating the PI3K pathway by binding directly to p110 [13]. Downstream of RAS, in the mitogen-activated protein kinase (MAPK) pathway, ERK has been shown to negatively regulate TSC2 [14]. Additionally, MAPK pathway activation has been identified as a consequence of mTORC1 inhibition, further intercalating these two important cascades [15].

## GENETIC ALTERATIONS IN THE PI3K PATHWAY IN CANCER

Deregulation of several elements of the PI3K signaling cascade is recognized in human cancer, the occurrence of which promotes pathway activation. The most prevalent are those affecting *PIK3CA* (the gene coding for p110α) and PTEN, as well as those affecting upstream RTKs. This latter group has been extensively reviewed previously and will not be discussed here.

Derangements in PTEN were the first described and are the most common abnormalities linked with PI3K signaling in human cancer. The *PTEN* gene maps to chromosome 10q23. Functional loss of PTEN impairs its lipid phosphatase activity, which is critical for its tumor suppressor function [16]. Reduced PTEN expression is found most commonly in endometrial, prostate, breast and ovarian cancers, as well as glioblastomas and melanomas. The somatic aberrations that affect PTEN (reviewed in [17]) can occur through allelic losses leading to either complete deletion of the *PTEN* locus, or point or truncating *PTEN* mutations resulting in functional inactivation. Epigenetic phenomena such as promoter methylation can also lead to gene silencing. Further, there are various regulators of PTEN transcription that can both upregulate (such as Myc and p53) and downregulate (such as NFκB) protein production, and miR-21 is the first identified microRNA that represses PTEN expression [18]. Finally, rare germline mutations at the *PTEN* locus result in a number of overlapping clinical conditions, including the autosomal dominant Cowden's syndrome, characterized by the presence of hamartomas and a susceptibility to cancer, especially those of the breast, thyroid and endometrium [19].

Genetic aberrations of *PIK3CA*, located on chromosome 3, are also commonly found in human cancer. Whereas mutations are most commonly described in breast, colorectal and endometrial cancers, as well as glioblastomas, gene amplification tends to occur with greatest frequency in cervical, gastric, lung, head and neck, and ovarian cancers [20]. The majority of mutations cluster in two hot spot regions in exon 9 (encoding the helical domain of p110α) and exon 20 (encoding the catalytic domain of p110α). Such hot spot changes have been shown to upregulate Akt and promote oncogenic transformation *in vitro* and *in vivo* [21,22]. The exon 9 mutations result in E545K and E542K amino acid substitutions and may affect interactions with regulatory proteins, including p85. On the other hand, the exon 20 mutation causes a H1047R alteration and may affect specificity or affinity of p110α towards its substrates [23]. It has been shown that to induce transformation, H1047R mutants depend on p85 binding whereas E545K and E542K mutants depend on RAS binding [24]. Precisely how *PIK3CA* amplifications affect PI3K activation is less clear.

Mutual exclusivity between mutations of PTEN and RAS, PI3K and RAS, and PTEN and p53 has been demonstrated in certain tumors [25-28]. In contrast, studies suggest functional PTEN loss and *PIK3CA* mutations can coexist in breast, endometrial and colon cancer, implying a level of non-redundancy, despite their opposing functions on phosphoinositides [29,30]. However, this is perhaps not so surprising given PTEN has non-PI3K dependent functions and that *PIK3CA* codes for only one isoform of p110, suggesting other isoforms may influence signaling. Indeed, there is a growing body of literature relating to the other isoforms. p110β and p110δ (class IA), and p110γ (class IB) have not been found to possess oncogenic mutations in human cancer. However, overexpression of the wild-type protein of these variants is transforming in cell culture, unlike their p110α cousin [31]. Further, those isoforms with predominant expression on white blood cells (p110δ and p110γ) appear to be important in hematological malignancies [32]. Another recently described finding of interest is that p110β drives tumorigenesis in certain cell-based models of PTEN loss [33].

Other elements of the PI3K pathway are also mutated in human cancer, albeit with lower frequency than *PIK3CA* mutation or PTEN loss. Mutations in *PIK3R1*, coding for the p85 regulatory subunit, are observed in a small proportion of colorectal and ovarian cancers. These mutations appear to relieve the inhibitory effect that p85 has on p110, resulting in overactivity of PI3K signaling [34,35]. Amplification of *AKT* is observed in a proportion of head and neck, gastric, pancreatic and ovarian tumors, whereas a missense mutation in the pleckstrin homology domain of *AKT1* has recently been described at low frequency in breast, colorectal and ovarian cancers [36-38].

## INHIBITORS OF THE PI3K/AKT/MTOR PATHWAY

Agents inhibiting the upstream RTKs are amongst the most established targeted therapies in oncology. This is particularly true for monoclonal antibodies (mAbs) directed against EGFR and HER2, both of which are RTKs that transduce signal at least in part through PI3K. Cetuximab (IgG1 chimeric mAb) and panitumumab (IgG2 fully human mAb) both target the extracellular domain of EGFR. Both are approved for use in colorectal cancer; cetuximab is also approved in head and neck cancers. Trastuzumab, a humanized IgG1 mAb that inhibits HER2, is used widely in the treatment of women with HER2-overexpressing breast cancer in both adjuvant and metastatic settings. Small molecule tyrosine kinase inhibitors against EGFR (gefitinib and erlotinib) and HER2 (lapatinib, which also targets EGFR) are also working their way into clinical use. However, here we will focus on the evolution of inhibitors that target elements further downstream of the RTKs in the PI3K pathway.

### mTOR inhibitors – the rapalogs

As part of the mTORC1 complex, mTOR stimulates cell growth and protein synthesis through effects on mRNA translation and ribosome biogenesis (reviewed in [10]). Rapamycin is a macrolide antibiotic originally derived from *Streptomyces hygroscopicus* found in the soil on the island of Rapa Nui. Rapamycin (and its analogues, also known as rapalogs) acts by binding to the FKBP12 binding protein, which in turn interacts with the mTORC1 complex, inhibiting downstream signaling [39]. Though the rapalogs trace their history back to use as immunosuppressant drugs used in transplant medicine, their antiproliferative effects led to investigation of their use as anti-cancer agents. The other rapalogs, synthetic derivatives of rapamycin with improved properties, are temsirolimus (CCI-779; Wyeth, Madison, NJ, US), everolimus (RAD001; Novartis, Basel, Switzerland) and ridaforolimus (AP23573, formerly known as deforolimus; Merck & Co., Whitehouse Station, NJ, US).

Despite the high expectation for their application in oncology based on sound rationale related to the presumed mechanism-of-action, the rapalogs have only met with modest success. Most notable is the utility of these agents as monotherapy in renal cell cancer (RCC) and mantle cell lymphoma.

In RCC, a phase III trial investigated temsirolimus, interferon or the combination of both in previously untreated poor-prognosis patients. Those randomized to receive the rapalog as monotherapy had a response rate (RR) of 8.6% and a significantly longer overall survival (OS) and progression-free survival (PFS) compared to the other two study arms, leading to US Food and Drug Administration approval for this indication [40]. A further phase III study of everolimus versus placebo in RCC where patients had progressed on vascular endothelial growth factor (VEGF) receptor TKIs (sunitinib or sorafenib) was also positive for PFS in favor of the rapalog [41]. There was no OS benefit, however 80% of patients who initially received placebo subsequently crossed-over to everolimus treatment, diluting any potential effect. Additionally, although the RR was low (1.8%), an impressive 25% of patients remained progression free for 10 months or greater. Temsirolimus has also been investigated in a phase III trial of refractory mantle cell lymphoma, where it demonstrated superior RR and PFS compared with the control arm (investigator's choice of therapy) [42]. The rapalogs have been investigated as monotherapy in a host of other phase II studies in diverse tumor types, including neuroendocrine tumors, breast cancer, endometrial cancer and sarcomas [43]. Encouraging single agent clinical efficacy was observed with the use of everolimus in pretreated patients with recurrent endometrial cancer, where loss of PTEN expression was predictive of clinical benefit [44].

Overall, the activity of rapalogs in a host of tumor types where the PI3K/Akt/mTOR pathway is frequently activated has been disappointing. As a general rule, these agents only inhibit the mTORC1 complex (although there are some cellular models where disruption of mTORC2 also occurs) [10]. Therefore, there have been legitimate concerns that there efficacy may be partly limited by a failure to stop mTORC2 mediated phosphorylation and activation of Akt. In addition, inhibiting mTORC1 releases the feedback inhibition mediated by the S6K-IRS1-PI3K loop that normally acts to moderate pathway activity. This can lead to a paradoxical increase in Akt activity that can have both biological and therapeutic implications. Indeed, increased phosphorylated Akt has been detected in tumor biopsies from patients treated with rapalogs [45]. Altogether, these data suggest that pathway activation and reactivation could be avoided by PI3K, Akt or concomitant PI3K and mTOR catalytic inhibition (that would target both mTORC1 and mTORC2).

## PI3K INHIBITORS

A series of compounds are currently passing through the early phases of clinical development (summarized in table 1). ‘Pure’ PI3K inhibitors target only p110; both pan-p110 inhibitors and isoform-specific inhibitors exist. As the catalytic domains of the p110 subunits and mTOR are structurally similar, dual inhibitors of both PI3K and mTOR and are also emerging. These dual inhibitors suppress mTOR in both the mTORC1 and mTORC2 complexes, distinct from the rapalogs. With few exceptions, these agents act in an ATP-competitive and reversible manner.

**Table 1:** Summary of presented results from PI3K inhibitors in phase I clinical trials BW – twice weekly; QD – once daily; BD – twice daily; 21/7 – 21 days on, 7 days off; CDD – continuous daily dosing; MTD – maximum tolerated dose; MAD – maximum administered dose; AST – aspartate transaminase; ALT – alanine transaminase; AE – adverse event; NHL – non-Hodgkin's lymphoma; MCL – mantle cell lymphoma; CLL – chronic lymphocytic leukemia.

	Dual PI3K/mTOR inhibitors				Pure PI3K inhibitors				
**Compound (Company)**	SF1126 (Semafore)	NVP-BEZ235 (Novartis)	XL765 (Exelixis-Sanofi)	GDC-0980 (Roche-Genentech)	NVP-BKM120 (Novartis)	XL147 (Exelixis-Sanofi)	PX-866 (Oncothyreon) - Irreversible inhibitor	GDC-0941 (Roche-Genentech)	CAL-101 (Calistoga) - p110δ isoform specific inhibitor
**No. of patients**	39	59	83	17	35	78	60	59	106
**Administration schedule**	Intravenous BW	Oral QD	Oral BD or QD	Oral QD 21/7	Oral QD	Oral, QD: 21/7 or CDD	Oral, QD: Intermittent or CDD	Oral QD or BD (21/7)	Oral BD or QD
**MTD (or MAD)**	1110mg/m2 (MAD)	1100mg (MAD)	50mg (BD) 90mg (QD)	16mg (MAD)	100mg	600mg (both schedules)	12mg (intermittent) 8mg (CDD)	245mg (QD) 180mg TDD (BD)	350mg (MAD BD), 300mg (MAD QD)
**DLTs**	Diarrhea	None	Rash, nausea, vomiting, ↓ PO4 / anorexia, transa-minitis (BD) Abnormal ECG, rash / fatigue, dyskinesia (QD)	None	Mood alteration, epigastralgia, rash, hyper-glycemia	Rash (21/7) Hyper-sensitivity (CDD)	Diarrhea, ↑ AST (intermittent) Diarrhea (CDD)	Headache, pleural effusion, ↓ DLCO	↑ AST / ALT
**AEs (most common)**	Nausea, vomiting, diarrhea, fever, fatigue	Fatigue, diarrhea, nausea, vomiting, anorexia	Nausea, diarrhea, anorexia, vomiting, transa-minitis	Nausea, fatigue, diarrhea, flatulence	Rash, hyper-glycemia, diarrhea, anorexia, nausea	Nausea, fatigue, diarrhea, rash, cough	Diarrhea, nausea, vomiting, ↑ AST/ALT, fatigue	Nausea, fatigue, diarrhea, dysguesia	↑AST/ALT, pneumonia, neutropenia, anemia, thrombocytopenia
**Best response (in evaluable patients)**	Stable disease	Partial response (2 pts)	Stable disease	Stable disease	Partial response (2 pts)	Partial response (1 pt)	Stable disease	Partial response (1 pt)	Partial response (31 pts) NHL 57% MCL 67% CLL 30%
**Reference**	[49]	[53]	[54]	[55]	[60]	[61]	[63]	[66]	[68]

The first generation PI3K inhibitors were Wortmannin and LY294002. Wortmannin is a fungal metabolite initially isolated from *Penicillium wortmanni* in 1957. LY294002, about 500 times less potent and first produced about 25 years ago, is a synthetic compound derived from quercetin, a broad-spectrum kinase inhibitor [46]. Both agents achieve significant growth inhibition across a broad spectrum of cancer cell lines especially in circumstances of excess PI3K activity. However, neither Wortmannin nor LY294002 have progressed to clinical trials due to unfavorable pharmacokinetic properties, poor selectivity and toxicity concerns [47]. Regardless, their use has led to a greater understanding of the PI3K pathway and has spawned a new generation of inhibitors that overcome some of the failings of these compounds (summarized in table 1).

### Dual PI3K-mTOR inhibitors

As mentioned, agents of this class target all catalytic isoforms of PI3K together with mTORC1 and mTORC2. This has the theoretical advantage of more completely shutting down the PI3K/Akt/mTOR pathway but also the possible drawback of greater toxicity.

SF1126 (Semafore Pharmaceuticals, Indianapolis, IN, US) is a small molecule prodrug of LY294002 that is conjugated to an integrin-binding component. This design enhances delivery to the tumor and its associated vasculature where cleavage leads to release of the active drug. It has shown significant anti-tumor effects in xenograft models of solid tumors including glioblastoma, breast and prostate cancer, and potent anti-angiogenic activity has also been observed, felt partly to be related to a reduction in HIF-1α levels [48]. A phase I trial of patients with solid tumors is ongoing. No maximum tolerated dose (MTD) has been found, but the maximum administered dose (MAD) has been declared at 1110mg/m^2^ as intravenous administration. The most frequent adverse events were gastrointestinal complaints, fever and fatigue; there were no clinically significant effects on glucose or insulin levels. No responses were observed, but 19 of 38 evaluable patients (50%) showed stable disease as best response, for a median of 13 weeks and a mean of 18 weeks [49].

Two dual inhibitors are under investigation by Novartis (Basel, Switzerland) – NVP-BEZ235 and NVP-BGT226 (there is currently no presented or published data relating to NVP-BGT226). NVP-BEZ235 is an orally available product belonging to the class of imidazoquinolines [50]. Preclinical studies demonstrated anti-proliferative activity against a wide range of cancer cell lines, including HER2-overexpressing breast cancer models of trastuzumab and lapatinib resistance [51,52]. Further, tumor growth suppression has been shown in PI3K mutated xenograft models of human cancer. First data from the phase I clinical trial of NVP-BEZ235 was presented at the 46th American Society of Clinical Oncology (ASCO) annual meeting (2010) [53]. No DLTs have been observed in the first 59 treated patients. Of the 51 evaluable patients, two achieved a partial response – an estrogen receptor (ER) positive, HER2 negative breast cancer patient with unknown PI3K pathway status; and a patient with Cowden's syndrome (germline *PTEN* mutation) who had developed lung cancer. A further 14 patients (27%) achieved stable disease for 4 months or greater.

XL765 (Exelixis, South San Francisco, CA, US), also known as SAR245409, is another dual inhibitor. Tumor stabilization or shrinkage has been observed with XL765 in a variety of mouse xenograft models of human cancer, including breast, ovary, lung, prostate and brain cancers. Updated clinical data from the phase I monotherapy study in patients with solid tumors has demonstrated stable disease in 12 patients for 16 weeks or more and in 7 patients for 24 weeks or more (of a total of 83 enrolled patients) [54]. The most frequently observed toxicities involved elevated liver enzymes, gastrointestinal complaints and rash. The MTD has been defined as 50mg twice daily or 90mg daily.

GDC-0980 (Genetech, South San Francisco, CA, USA), also a PI3K/mTOR inhibitor, is under evaluation in a phase I clinical study of patients with solid tumors [55]. Though the study is in its earlier stages compared to those above, initial results show it to be well tolerated with no DLTs, and some suggestions of anti-tumor activity.

Other dual PI3K-mTOR inhibitors in clinical development include the orally administered PF-04691502 (Pfizer, New York, NY, US), and an intravenous agent, PKI-587 or PF-05212384 (Pfizer, New York, NY, US). Based on preclinical studies, phase I clinical trials are underway to assess safety and tolerability of these drugs in cancer patients with solid tumors [56,57].

### Pure PI3K inhibitors

The majority of compounds described as pure PI3K inhibitors are pan-p110 inhibitors. However, at least one isoform-specific inhibitor (CAL-101) has had preliminary results presented.

NVP-BKM120 (Novartis, Basel, Switzerland) is one such agent, and preclinical data showed anti-tumor activity in xenograft models of human cancer both with and without PI3K/PTEN mutations [58,59]. Preliminary results from the phase I study of NVP-BKM120 in patients with solid tumors were also presented at the 46th ASCO annual meeting [60]. Interestingly, though hyperglycemia has been an anticipated adverse event when using agents that inhibit the PI3K pathway due to its influence on cellular metabolism and insulin/glucose regulation, NVP-BKM120 is the only inhibitor in clinical trials that has encountered clinically relevant elevations in plasma glucose. Indeed, hyperglycemia was a DLT, as was mood alteration and rash. The MTD was identified as 100mg daily. Of the 31 evaluable patients, there were two partial responses. Both were in women with breast cancer – one had a triple negative breast cancer (ER and progesterone receptor (PR) negative, HER2 negative) that was *PIK3CA* wild type, without PTEN loss and *KRAS* mutant; and the other had a ER/PR positive, HER2 negative tumor with a confirmed *PIK3CA* mutation (E545K). Additionally, 20% of patients remained on study for at least 8 months.

XL147 (Exelixis, South San Francisco, CA, US), also known as SAR245408, is another pan-p110 inhibitor. It has shown preclinical activity in a variety of xenograft models of human cancer, including those of breast, lung and prostate cancer. Initial data from the first 60 patients treated with this agent as monotherapy in a phase I study was presented at the same ASCO meeting [61]. Rash was the DLT, setting the MTD at 600mg on either an intermittent (21 out of 28 days) or continuous daily dosing schedules, with fatigue, nausea, vomiting and diarrhea also attributable to the drug. Of the patients evaluable for response, there was a partial response in a non-PI3K/PTEN mutated non-small cell lung cancer patient, and 19% of patients continued on treatment for a minimum of 16 weeks.

The semisynthetic wortmannin derivative PX-866 (Oncothyreon, Seattle, WA, US), also a pan-isoform inhibitor of class I PI3Ks, differs from other agents targeting PI3K in that it covalently binds to the ATP-binding site of p110 and is thus irreversible. *In vivo* studies demonstrate that *PIK3CA* mutant or PTEN null xenografts were sensitive to treatment with PX-866 [62]. Final results from 60 patients treated on the phase I study of PX-866 have been presented [63]. The MTD was defined as 8mg and 12mg on the continuous and intermittent schedules, respectively, with DLTs of diarrhea and elevated liver enzymes. Nausea, vomiting and fatigue were also amongst the more common adverse events seen. No responses were seen amongst the 53 evaluable patients, but 25% of these heavily pretreated patients achieved stable disease for a median of 57 days.

PI103 was one of the earlier new generation PI3K inhibitors that showed proof-of-concept whereby targeting members of the PI3K family with high selectivity was able to achieve target modulation with resultant *in vivo* antitumor activity [64]. Its rapid metabolism precluded clinical development, but proved a valuable tool that ultimately led to development of GDC-0941 (Piramed/Genentech, Slough, United Kingdom/South San Francisco, CA, US) another pan-isoform class I PI3K inhibitor. This derivative of thieno[3,2-d]pyrimidine has demonstrated tumor growth inhibition in xenograft models including those harboring mutations in PI3K or PTEN [65]. In a phase I study of GDC-0941 administered as monotherapy, the most frequently reported drug-related adverse events were mild or moderate nausea, fatigue, diarrhea, and dysgeusia [66]. The three DLTs reported were headache, pleural effusion and decreased lung diffusion capacity. One partial response has been observed in a breast cancer patient, and encouraging activity has also been seen in patients with ovarian cancer.

Finally, there has been interest in developing isoform-specific inhibitors as it may permit more complete target inhibition with a more tolerable adverse effect profile. The most advanced is a p110δ-specific inhibitor – CAL-101 (Calistoga Pharmaceuticals, Seattle, WA, US). The p110δ isoform is expressed predominantly in leukocytes, and preclinical work showed it to be efficacious in lymphoma and leukemia cells and promoted apoptosis [67]. Accordingly, 106 patients with chronic lymphocytic leukemia (CLL), different types of non-Hodgkin's lymphoma (NHL), acute myeloid leukemia (AML) and multiple myeloma (MM) have been enrolled thus far into a phase I study of CAL-101 [68]. Reversible increases in liver enzymes and pneumonia have been the most frequent treatment emergent adverse events, although there was minimal hematological toxicity. Impressively, partial responses have been seen in 13 of 23 patients (57%) with indolent forms of NHL, 8 of 12 patients (67%) with mantle cell lymphoma and 10 of 30 patients (33%) with CLL.

### Akt inhibitors

Direct inhibition of the serine/threonine kinase Akt provides another avenue to pharmacologically regulate activity of the PI3K pathway. The two strategies being explored involves agents that compete for the ATP-binding site (ATP mimetics) and those that act away from this catalytic site (allosteric inhibitors). As is the case with PI3K inhibitors, there is some expectation that tumors harboring mutations or amplifications of Akt, or increased pathway activity, will show greater sensitivity to Akt inhibitors. However, as with the rapalogs, the release of feedback inhibition consequent to targeting Akt may enhance the activity of non-Akt effectors of PI3K signaling. Further, these non-Akt dependent effectors of PI3K signaling, such as SGK3, can promote cancer in the presence of *PIK3CA* mutations [69]. Despite these findings, a recent study demonstrated that a noncatalytic site Akt inhibitor was effective against breast cancer cell lines with *PIK3CA* mutations and *HER2* amplifications [70]. In addition, another study demonstrated that tumors with *PIK3CA* mutations were the most sensitive to an Akt plekstrin homology (PH) domain inhibitor, and *KRAS* mutant tumors were the least sensitive [71].

Perifosine (Keryx Biopharmaceuticals, New York, NY, US) is an allosteric inhibitor that targets the PH domain of Akt, thereby preventing its translocation to the plasma membrane required for activation [72]. It exerts Akt-dependent and Akt-independent effects, and although many preclinical studies have documented Akt inhibition by perifosine, clinical validation of these findings is lacking [73]. Perifosine has been evaluated in a host of phase I/II clinical trials both as monotherapy and in combination with various other agents. The most common adverse reactions are fatigue and gastrointestinal toxicity. The latter led to frequent treatment discontinuation; alterations to the dosing schedule helped rectify this problem [74]. Single-agent activity with perifosine has generally been disappointing, although activity has been observed in patients with sarcoma and Waldenström's macroglobulinemia [75,76].

MK-2206 (Merck & Co., Whitehouse Station, NJ, US) is another allosteric Akt inhibitor. In preclinical studies, synergism has been demonstrated when MK-2206 has been used in combination with other targeted therapies (erlotinib, lapatinib) or a host of cytotoxic agents [77]. Preliminary results of a phase I study in solid tumors have been presented [78]. The MTD has been defined as 60mg and 200mg on the daily and weekly schedules, respectively. DLT was rash, with other common side effects being fatigue and gastrointestinal complaints. No patient achieved a partial response, although tumor shrinkage of up to 23% was seen in several patients, especially those with pancreatic cancer (both adenocarcinoma and neuroendocrine histologies).

GSK690693 (GlaxoSmithKline, Brentford, UK) is a potent ATP-competitive Akt inhibitor that also inhibits the phosphorylation of the downstream target GSK3 in cells. It is currently in clinical development as an intravenous agent for use in patients with solid tumors or hematological malignancies.

Other orally dosed Akt inhibitors undergoing phase I first-in-human trials in cancer patients include GSK2141795 (GlaxoSmithKline, Brentford, UK), GSK2110183 (Octagon Research Solutions, Wayne, PA, US), GDC-0068 (Genentech, South San Francisco, CA, US), and LY2780301 (Eli Lilly and Company, Indianapolis, IN, US).

### mTOR kinase inhibitors

A new variety of mTOR inhibitor has recently emerged. They are ATP-competitive inhibitors and thus target the kinase domain of mTOR, repressing both mTORC1 and mTORC2 activity. Therefore, they share more in common with the dual PI3K/mTOR inhibitors than the rapalogs in terms of their mechanism-of-action. In turn, this should mitigate the paradoxical PI3K activation consequent to de-repression of the negative feedback seen with rapalogs. Despite this advantage, interesting preclinical data of two such agents (PP242 and PP30) suggests that they have more substantial antiproliferative actions than rapamycin not because of the mTORC2 effects but rather because they are more effective in suppressing mTORC1 [79]. Other agents in this group include WAY-600, WYE-687, and WYE-354, the latter of which has displayed robust antitumor activity in PTEN-null tumor xenografts [80].

AZD8055 (Astra Zeneca, London, UK), OSI-027 (OSI Pharmaceuticals, Melville, NY, US) and INK128 (Intellikine, La Jolla, CA, US) are the first mTOR kinase inhibitors to enter clinical trials [81]. Preliminary data from a phase I trial of OSI-027 was presented at the 46th ASCO annual meeting [82]. Only 43 patients have been treated across 3 dosing schedules thus far. DLTs of fatigue and a decrease in cardiac left ventricular ejection fraction have been noted, but the most common side effects have been fatigue, anorexia and nausea. Stable disease has been the best response to date, although tumor shrinkage has been seen in a patient with colorectal cancer and another with a parotid adenoidcystic cancer.

## BIOMARKERS

Biomarker studies are becoming increasingly incorporated into early phase clinical trials. This is largely true for the phase I trials of PI3K pathway inhibitors described above where various predictive and pharmacodynamic (PD) biomarkers have been explored. PD biomarkers are markers of drug effect that assess for target inhibition and pathway downregulation. They necessitate assessment prior to and following an intervention to detect a change from baseline; a correlation with clinical activity is not implied but is desirable. A number of different biological tissues have been acquired from patients on these trials in order to perform these biomarker studies (summarized in table 2). Predictive biomarkers predict the efficacy (or lack thereof) of a particular treatment in a given clinical scenario (discussed below).

**Table 2:** Summary of presented pharmacodynamic biomarker studies from phase I clinical trials of inhibitors of the PI3K/Akt/mTOR pathway PD – pharmacodynamic; PBMC – peripheral blood mononuclear cell; FDG-PET – fluorodeoxyglucose positron emission tomography.

Drug	Target/s	Acquired PD biomarker					Comments / findings
		Skin	Hair	PBMC / plasma	Tumor	FDG-PET	
SF1126	PI3K/mTOR	✓			✓		↓ pAkt^S473^ & ↑ apoptosis in circulating lymphocytes (selected cases)
NVP-BEZ235	PI3K/mTOR			✓	✓	✓	Dose-dependent ↑ in plasma C-peptide (no significant ↑ glucose) ↓ pS6 & ↑Ki67 in tumor biopsies (selected cases) ↓ in FDG-PET uptake in 18 of 37 (49%) pts
XL765	PI3K/mTOR	✓	✓	✓	✓	✓	Modest ↑ in plasma insulin (no effect on glucose) PI3K pathway inhibition in hair & skin across doses including MTD Robust PI3K pathway inhibition across diverse tumor types: - ↓ pAkt^S473^ (50–90%), ↓ pAkt^T308^ (50–80%), ↓ p4EBP1 (60–90%) MAPK pathway inhibition in tumors: ↓ pERK (40–80%)
GDC-0980	PI3K/mTOR			✓		✓	↓ pAkt in platelet rich plasma
NVP-BKM120	PI3K	✓		✓		✓	↑ in C-peptide (with associated ↑ glucose) ↓ pS6 in skin (40–85% in most pts treated at 80–150mg doses) ↓ in FDG-PET uptake in most pts (≥ 25% in 10 pts)
XL147	PI3K	✓	✓	✓	✓	✓	Minor ↑ in plasma insulin (no effect on glucose) PI3K pathway inhibition in hair & skin across doses including MTD Robust PI3K pathway inhibition across diverse tumor types: - ↓ pAkt^T308^ (40–80%), ↓ p4EBP1 (60–90%) MAPK pathway inhibition in tumors: ↓ pERK (40–60%)
PX-866	PI3K			✓	✓		PI3K pathway inhibition in PBMCs (pS6 and p-mTOR)
CAL-101	PI3K - p110δ specific				✓		Robust PI3K pathway inhibition in CLL cells: ↓ pAkt^T308^ (70–90%)
MK-2206	Akt			✓	✓		PI3K pathway inhibition in whole blood pAkt at all dose levels Robust PI3K pathway inhibition in tumor: ↓ pAkt up to 90% in 5/7 paired samples
OSI-207	mTOR (mTORC1 & mTORC2)			✓			mTOR pathway inhibition in PBMCs: ↓ p4EBP1 (>60% in most patients)

In the clinical trials of PI3K inhibitors where preliminary PD outcomes have been reported, diminution in pathway readouts has been observed, giving reassurance that the target is being hit. For example, the XL765 and XL147 studies had an extensive biomarker component. Results have shown reduced activation of key pathway nodes in the order of 50–90% in both tumor and non-tumor tissue [54,61]. However, this does not necessarily equate with meaningful clinical benefits. Regardless, translational research requires biomarker studies to further knowledge and to assist in finding solutions to clinical problems or disappointments, and often raises new questions of interest. Indeed, the reduction in pERK (a marker of MAPK pathway activity) noted in tumors of patients treated with XL765 and XL147 was unexpected, raising the possibility of hitherto unrecognized crosstalk between the PI3K and MAPK pathways [54,61].

At present, an important concern is that many biomarker assays have been neither standardized nor validated. They add to the cost of the trial and may involve invasive procedures that carry a degree of risk to the patient. Evaluation of PTEN status is a prime example. Because functional PTEN loss can occur through a variety of mechanisms, detection of PTEN protein expression by immunohistochemistry (IHC) on tumor samples is the preferred method. However, the antibodies used to stain samples are not uniform between laboratories, nor has a definitive cut-off been defined below which PTEN is considered to be lost. Further, the adequacy of archival compared to fresh tissue has not been delineated. And given that tumor samples are often small and difficult to obtain, how biomarker studies ought to be prioritized is not clear.

One solution is to find adequate surrogate markers. Imaging modalities provide an option. Patients on the NVP-BKM120 trial underwent FDG-PET scans. Reduced PET avidity in was seen in lesions of most patients [60]. This seems encouraging, but whether it represents true anti-cancer activity or merely the impact that PI3K inhibition has on glucose homeostasis remains to be seen. Biomarkers detectable in peripheral blood have the advantage of being minimally invasive and accessible for repeat samples. Mechanism-based toxicities of PI3K/Akt/mTOR inhibitors that could potentially be used as PD biomarkers include hypertriglyceridemia and hyperglycemia [83]. The NVP-BZ235 and BKM-120 trials found an increase in plasma C-peptide levels following treatment as a surrogate for the insulin resistance anticipated from pathway inhibition [53,60]. Also, a reduction in pAkt was seen in platelet-rich plasma obtained from patients treated with GDC-0980 [55]. These are promising examples, but require further analysis. Regardless, provided biomarker studies are employed with careful forethought and selectivity, their place in clinical trials is justified.

## FUTURE STRATEGIES

The preliminary clinical data from phase I trials presented to date have not demonstrated significant response rates with any of the inhibitors when employed as single agent therapy. The potential reasons for this finding include poor patient selection, inadequate dosing schedules, and resistance mechanisms.

Regarding patient selection, strong preclinical work has suggested that those patients whose tumors harbor genetic aberrations that result in increased PI3K pathway activity should be most sensitive to these agents [52,62,70,84]. Indeed, many of the clinical studies have retrospectively analyzed pathway genetics sourced from archival or fresh tumor tissue (in particular, but not restricted to, PTEN and *PIK3CA* status). However, the majority of patients with detected PTEN loss or *PIK3CA* mutations have not responded to monotherapy. In addition, the few confirmed clinical responses seen have occurred in both those with and those without PI3K pathway activating mutations. Nonetheless, it seems a reasonable strategy to enrich patient populations with those harboring such genetic changes and prospective analysis of these potential predictive biomarkers should be employed.

A second area of contention relates to dosing schedules. PD biomarker studies have shown robust PI3K pathway inhibition following treatment but complete pathway shutdown is not achieved. There is ongoing discussion regarding whether this is an inadequate strategy. Intermittent dosing schedules employing higher doses for shorter durations (thus potentially minimizing the risk of cumulative toxicity) may boost the clinical outcomes if 100% pathway inhibition can be attained.

A third strategy that is well underway is the use of drug combinations. Signaling pathways in human cancer are complex. Frequent cross-talk and feedback loops add to complexity and promote avenues for resistance. Except for the relatively uncommon scenario of genuine oncogenic addiction, it seems unlikely that blocking a single pathway will be sufficient to switch off the drive for malignant growth and progression in a tumor. There is much optimism that use of rationale drug combinations should overcome some of these deficiencies. This could imply any of the drug classes described here co-administered with either targeted therapies against RTKs, key nodes in parallel pathways, or cytotoxic agents.

The rapalogs have shown early encouraging data. PI3K pathway activation has been found to lead to resistance to trastuzumab in HER2-overexpressing breast cancer [52]. Accordingly, studies have investigated adding everolimus to trastuzumab and paclitaxel in women with prior resistance to the latter two agents. Confirmed partial responses were seen in 20% of subjects and stable disease in a further 56% in a phase II study [85]. The same strategy has been evaluated in a phase I trial of everolimus, trastuzumab and vinorelbine, achieving a disease control rate of 80% (37 of 46 evaluable patients) [86]. The combination of a rapalog (ridaforolimus) and a monoclonal antibody targeting the IGF1-R (dalotuzumab, MK-0646) has been studied in a phase I trial of patients with solid tumors [87]. Stomatitis was the DLT. Importantly, partial responses were seen in 6 of 62 patients (10%), despite the relatively poor response rates of either agent as monotherapy, supporting the notion that combinations can lead to better outcomes. There are many more combinations with rapalogs currently under evaluation.

Amongst the PI3K pathway inhibitors, a host of phase I studies evaluating combination strategies are underway. As seen in table 3, co-administration with either molecular targeted therapies, as well as cytotoxic agents, is being evaluated. Finally, there is some evidence showing that inhibition of the PI3K pathway can lead to hyperactivation of the MAPK pathway, and hence combinations of PI3K inhibitors and MEK inhibitors may be a promising therapeutic strategy.

**Table 3:** Phase I clinical trials of PI3K pathway inhibitors in combination with targeted agents and chemotherapeutics Legend: HER2 - human epidermal growth factor receptor 2; HR - hormone receptor; NHL – non-Hodgkin's lymphoma; CLL – chronic lymphocytic leukemia.

Target	Agent	Study Population	Combination	Clinical Trial
**PI3K/mTOR**	NVP-BEZ235	Advanced breast cancer HER2 +	Trastuzumab	NCT00620594
	XL765	Advanced solid tumors and non-small cell lung cancer	Erlotinib	NCT00777699
		High-grade gliomas	Temozolomide	NCT00704080
**pan-PI3K**	NVP-BKM120	Advanced breast cancer HER2 +	Trastuzumab	NCT01132664
		Advanced solid tumors with RAS/RAF mutations and triple negative breast cancer	GSK1120212 (MEK inhibitor)	NCT01155453
	XL147	Advanced solid tumors	Erlotinib	NCT00692640
		Advanced solid tumors	Paclitaxel and Carboplatin	NCT00756847
		Advanced breast cancer HER2 +	Trastuzumab or Paclitaxel and Trastuzumab	NCT01042925
		Advanced breast cancer HR +	Letrozole	NCT01082068
	GDC-0941	Advanced solid tumors and non-small cell lung cancer	Erlotinib	NCT00975182
		Advanced non-small cell lung cancer	Paclitaxel and Carboplatin with or without Bevacizumab	NCT00974584
		Advanced breast cancer HER2 +	Trastuzumab-MCC-DM1	NCT00928330
		Advanced breast cancer	Paclitaxel and Bevacizumab	NCT00960960
		Advanced solid tumors	GDC-0973 (MEK inhibitor)	NCT00996892
**PI3K - p110δ specific**	CAL-101	Indolent B-cell NHL or CLL	Bendamustine and Rituximab	NCT01088048
**Akt**	MK-2206	Advanced solid tumors	Paclitaxel and Carboplatin or Docetaxel or Erlotinib	NCT00848718
		Advanced breast cancer HER2 +	Trastuzumab and Lapatinib	NCT00963547
		Advanced solid tumors	AZD6244 (MEK inhibitor)	NCT01021748

## CONCLUSION

The rapalogs provide one avenue for inhibiting the PI3K/Akt/mTOR pathway. They have had some success but left much room for improvement. As the newer agents progress through clinical evaluation – inhibitors of PI3K, Akt, and mTOR kinase inhibitors – the early findings suggest the drugs are relatively well tolerated and that pathway downregulation is being achieved. However, there have been relatively few clinical responses, even amongst those patients with PTEN loss or activating mutations of PI3K. Irrespective, investigators are devising and employing new strategies to enhance outcomes, in particular by enriching patient populations and testing a multitude of drug combinations based on sound rationale. In addition, agents targeting other components of the pathway are under development. These include PDK1 inhibitors (to prevent Akt^T308^ phosphorylation and non-PI3K dependent phosphorylation of other kinases that can promote cancer progression), SHIP agonists (to promote PIP3 degradation), and heat shock protein inhibitors (Akt is a client protein of the molecular chaperone). Given the importance of the PI3K pathway in the malignant phenotype, further optimization of the clinical use of these new compounds in the coming years is warranted and should lead to better patient outcomes.
